# Binding determinants in the interplay between porcine aminopeptidase N and enterotoxigenic *Escherichia coli* F4 fimbriae

**DOI:** 10.1186/s13567-018-0519-9

**Published:** 2018-02-26

**Authors:** Pengpeng Xia, Guomei Quan, Yi Yang, Jing Zhao, Yiting Wang, Mingxu Zhou, Philip R. Hardwidge, Jianzhong Zhu, Siguo Liu, Guoqiang Zhu

**Affiliations:** 1 College of Veterinary Medicine, Yangzhou University, Yangzhou, 225009 China; 2 Jiangsu Co-innovation Center for Prevention and Control of Important Animal Infectious Diseases and Zoonoses, Yangzhou, 225009 China; 3 National Key Laboratory of Veterinary Biotechnology, Harbin Veterinary Research Institute, Chinese Academy of Agricultural Sciences, Harbin, 150001 China; 4 College of Veterinary Medicine, Kansas State University, Manhattan, KS 66506 USA; 5 Joint International Research Laboratory of Agriculture and Agri-Product Safety of Ministry of Education of China, Yangzhou University, Yangzhou, 225009 China

## Abstract

The binding of F4^+^ enterotoxigenic *Escherichia coli* (ETEC) and the specific receptor on porcine intestinal epithelial cells is the initial step in F4^+^ ETEC infection. Porcine aminopeptidase N (APN) is a newly discovered receptor for F4 fimbriae that binds directly to FaeG adhesin, which is the major subunit of the F4 fimbriae variants F4ab, F4ac, and F4ad. We used overlapping peptide assays to map the APN-FaeG binding sites, which has facilitated in the identifying the APN-binding amino acids that are located in the same region of FaeG variants, thereby limiting the major binding regions of APN to 13 peptides. To determine the core sequence motif, a panel of FaeG peptides with point mutations and FaeG mutants were constructed. Pull-down and binding reactivity assays using piglet intestines determined that the amino acids G159 of F4ab, N209 and L212 of F4ac, and A200 of F4ad were the critical residues for APN binding of FaeG. We further show using ELISA and confocal microscopy assay that amino acids 553–568, and 652–670 of the APN comprise the linear epitope for FaeG binding in all three F4 fimbriae variants.

**Electronic supplementary material:**

The online version of this article (10.1186/s13567-018-0519-9) contains supplementary material, which is available to authorized users.

## Introduction

F4^+^ enterotoxigenic *Escherichia coli* (ETEC) infections cause neonatal and post-weaning diarrhea (PWD) in piglets, which are common gastrointestinal diseases affecting the global swine industry [1]. F4^+^ ETEC has three variants, namely, ab, ac, and ad. A comparative analysis of the gene cluster between these three variants show that their differences mainly involve the *faeG* gene, thereby causing variations in the adhesive properties and specificities of the F4 fimbriae [2, 3]. The major FaeG subunit is an essential component of F4 fimbriae. For instance, oral administration of F4 fimbriae or the FaeG protein induces a protective mucosal immune response [4, 5]. Moreover, the FaeG deleted strains show a significant reduction in adherence to host epithelial cells [6].

F4 receptor (F4R)-positive piglets are susceptible to F4^+^ *E. coli* bacterial infections, whereas F4R-negative piglets are resistant [7]. Thus, interactions between F4ab, F4ac, or F4ad fimbriae and specific receptors on the host intestinal are essential in initiating attachment, colonization, and infection of the three serotypes. A polymorphism within *mucin 4 intron 7* has been linked to an importance percentage of F4 ETEC adhesive phenotype and has been used in the primary selection of F4ab/ac-susceptible or -resistant piglets in various breeds [8, 9]. The inheritance pattern for the F4ad receptor is presumably controlled by a new mode; the partially adhesive receptor is dominated by only one gene and the fully adhesive receptor is encoded by more than one epistatic gene [10]. The receptor protein of the three variants also varies, i.e., the 210- and 240-kDa intestinal mucin-type glycoprotein interact with both F4ab and F4ac, whereas a 74-kDa glycoprotein (GP74) and an intestinal neutral glycosphingolipid specifically adheres to F4ab and F4ad, respectively [11].

Aminopeptidase N (APN) is a widely expressed membrane-bound exopeptidase that belongs to a group of zinc-containing metalloproteases that include the consensus catalytic motif HEXXH [12]. APN can activate or inactivate bioactive peptides on the cell surface, and cause cytokine and extracellular matrix degradation to show enzymatic activity. APN plays a role in inflammatory and immunological responses, antigen processing, tumor invasion, and cell–cell contact. Moreover, APN modulates signals in monocytes [13] and is a receptor for several coronaviruses, i.e., canine coronavirus, feline infectious peritonitis virus, and transmissible gastroenteritis virus (TGEV) [14, 15]. Importantly, APN is also a direct receptor for ETEC F4 fimbriae and is associated with the induction of mucosal immunity [16]. FaeG from all three variants directly mediate the fimbrial binding of F4^+^ *E. coli* to host intestinal epithelial cells by binding to APN, while also modulating APN expression in IPEC-J2 cells that in turn influences ETEC adherence [6, 17].

The present study has characterized the molecular details regarding APN-FaeG interactions, determined the binding sites in the interplay between APN and F4 fimbriae, and established whether APN interacts with the three variants at the same region.

## Materials and methods

### Bacterial strains, antibodies, cell lines, and culture conditions

Three serotypes of F4^+^ *E. coli* (C83901, O8:K87:F4ab; C83902, O8:K87:F4ac; and C83903, O141:K85:F4ad) strains were grown in Luria–Bertani (LB) media (Solarbio, Beijing, China), and three engineered serotypes of rF4 *E. coli* (SE5000 carrying vector PBR322-*faeG* expressing FaeG of F4ab, F4ac, and F4ad, respectively) [6] and the engineered bacteria harboring the pGEX-6p-1-FaeG plasmids expressing soluble FaeG protein from F4ab, F4ac, and F4ad were cultivated in LB media supplemented with 100 µg/mL ampicillin with continuous agitation (178 rpm) at 37 °C.

Porcine neonatal jejunal IPEC-J2 cells and stable transfected cell lines pEC129-APN IPEC-J2 and pcDNA™6.2-GW/miR-APN IPEC-J2 (knocked down APN expression) were grown in RPMI 1640-F12 (1:1) (Gibco, Australia) supplemented with 10% fetal bovine serum (FBS, Gibco, Australia) at 37 °C in a humidified incubator with an atmosphere of 6% CO_2_ [17]. We developed the monoclonal anti-F4 antibody and the polyclonal anti-APN antibodies in our laboratory [18, 19].

### Construction of recombinant plasmids

We used the recombinant plasmid pET28a(+)-APN as a DNA template for subsequent PCR amplification [17]. Five pairs of primers specific to the porcine APN mRNA (GenBank Accession Number: KF280271) were designed to amplify five truncated APNs, including primers for APN∆1 (401–963 amino acids (AA), forward: TTCATATGCAACCTGGTG, reverse: CGGAATTCGCTGTGCTCTA), APN∆2 (1–400 AA, forward: TTCATATGGCCAAGGGATTC, reverse: CGGAATTCGGTCACCAGGTTG), APN∆3 (332–400 AA, forward: TTCATATGTGCCGGTGCCA, reverse: CGGAATTCAGGGTCACCAG), APN∆4 (1–331 AA, forward: TTCATATGGCCAAGGGATTC, reverse: CGGAATTCAAGTCGGGCAAGG), and APN∆5 (332–963 AA, forward: TTCATATGTGCCGGTGCCA, reversed: CGGAATTCGCTGTGCTCTA). We sequenced the resulting PCR products, cloned these into pET-28a (+), and then transformed them into *E. coli* BL21 (DE3) cells for protein expression [20].

### Analysis of protein–protein interactions using enzyme-linked immunosorbent assay (ELISA)

We expressed GST-tagged FaeG fusion proteins from pGEX-6p-1-FaeG F4ab, pGEX-6p-1-FaeG F4ac, and pGEX-6p-1-FaeG F4ad, respectively, then analyzed the interaction between each of the five purified truncated APNs and FaeG using indirect ELISA. Specifically, we coated the APN and its five truncated proteins on ELISA plates at a final concentration of 100 μg/mL (100 μL/well) in a carbonate-bicarbonate buffer (15 mM Na_2_CO_3_, 35 mM NaHCO_3_, pH 9.6) at 4 °C overnight, washed the wells three times with PBST, incubated the wells in 5% bovine serum albumin (BSA, Sigma, USA) in PBST at 37 °C for 2 h, then sequentially washed the wells three more times in a PBST buffer, and incubated the wells in 100 μg/mL (100 μL/well) FaeG fusion protein. We used pGEX-6p-1 and pet28a (+) as negative controls. After washing the plates four times in PBST, we incubated the plates in an anti-F4 monoclonal antibody (1:1000 dilution in PBS) at 37 °C for 1 h, followed by HRP-conjugated goat anti-rabbit IgG (Abcam, USA) for 30 min [21]. The absorbance was read at a wavelength of 450 nm, and all samples were run in triplicate.

### Overlapping peptide assay

We designed two peptide libraries to represent and cover the entire APN coding region (963 AAs) and FaeG of the three variants (284 AAs for F4ac, and 286 AAs for both F4ab and F4ad). Each peptide was 13 amino acids in length and offset from its neighboring peptide by three amino acids. We automatically performed synthesis on an amino-PEG500-grafted cellulose membrane according to standard SPOT synthesis protocols [22, 23]. We activated the membranes with 318 spots for APN and 205 spots for FaeG in ethanol (every 5 min, thrice). Afterwards, we sequentially washed the membranes thrice times in a TBST buffer, blocked the membranes in 5% BSA-TBST, incubated these with bait proteins (1 mg/mL purified soluble proteins), and then blotted these with a monoclonal antibody against F4^+^ and polyclonal antibodies against APN, respectively, with BSA-TBST as negative control. The resulting chemiluminescent signals were analyzed using TotalLab 1D software (USA). The peptide with the strongest signal intensity was set to 100%, and the other spots were normalized to this value [23]. Signal with intensity values > 30% were considered positive. Tables 1 and 2 show the positive spots.

**Table 1:** **Analysis of polypeptide sequences in the positive spots on the APN membrane**

Spots	Sequences
10–11 (28–43 AA)	ALSVVYAQEKNKNAEH
18–20 (52–70 AA)	TITTTAAITLDQSKPWNRY
45–46 (133–148 AA)	VLRGVGDSQVPEIDRT
70–72 (208–223 AA)	QSTDARKSFPCFDEPA
76–78 (226–244 AA)	ATFNITLIHPNNLTALSNM
123–124//126–127 (367–391 AA)	QSSSISNKERVVTVIAHELAHQWFG
146–149 (436–457 AA)	YRVMAVDALASSHPLTTPAEEV
157–161 (469–493 AA)	SISYSKGASVIRMLSNFLTEDLFKE
185–186 (553–568 AA)	KTGNISQKHFLLDSES
194–196 (580–598 AA)	WIVPISSIKNGVMQDHYWL
218–220 (652–670 AA)	VINRAQVIYDSFNLATAHM
281–282 (841–856 AA)	YLGYTLNPDLIRKQDA
301–302 (901–916 AA)	GVTRRFSSEFELQQLE

**Table 2:** **Analysis of polypeptide sequences in the positive spots on the FaeG membrane**

Spots	Sequences
50 (148–160 AA)	NASYAGVFGKGGV
59 (175–187 AA)	LRAIFYGGLTTTV
67 (199–217 AA)	AARTELFGSLSRNDILGQI
116 (149–161 AA)	NASYAGVLGRGGV
125 (176–188 AA)	LSSIFYGGLPRGS
133–135 (200–218 AA)	TKLFGSLSRNDILGQIQRV
172 (149–161 AA)	ASYAGALGRGGVT
181 (176–188 AA)	HAIFYGGLPTNVK
189–191 (200–218 AA)	ARTELFGSLSKNDILGQIQ

### Confocal microscopy

The peptides used in this study were synthesized using Scilight-Peptide (Beijing, China) (Table 3). We harvested and deposited pEC129-APN IPEC-J2 cells onto a glass slide (NEST, Shanghai, China) overnight at 37 °C. We incubated cells of 60–70% confluency with polyclonal antibodies against APN for 1 h at 37 °C, then washed the slides thrice in warm PBS, followed by incubating with peptides (1 µM) for 30 min at room temperature. The FaeG protein was used as positive control, and pcDNA™6.2-GW/miR-APN IPEC-J2 and PBS were employed as negative controls. After incubating the slides in 20 μg/μL Dylight 549 goat anti-rabbit IgG (Univ-bio, Shanghai, China) for 30 min, these were washed in warm PBS, and then fixed in 4% pre-cooling paraformaldehyde for 20 min. The slides were dried and then stained with DAPI (TianGen, Beijing, China) for 10–15 min, washed and mounted with cover slips. Images were captured using a Leica TCS SP8 STED confocal microscope (Wetzlar, Germany). To further investigate the critical residues in the linear epitope, we analyzed the binding between the purified FaeG and APN proteins and their peptides using ELISA [21]. We synthesized the 13 peptides of APN and coated these on ELISA plates at a final concentration of 10 μg/mL (100 μL/well), then incubated the wells with 10 μg/mL (100 μL/well) FaeG peptides or fusion proteins. We used non-binding peptide (MSPILGYWKIKGL, amino acids of the GST-tag) as negative control. We recorded the absorbance at a wavelength of 450 nm, and ran all of the samples in triplicate.

**Table 3:** **Peptides used in this study**

Peptides	Sequences
APN 28–43	FITC-Ahx-ALSVVYAQEKNKNAEH
APN 52–70	FITC-Ahx-TITTTAAITLDQSKPWNRY
APN 133–148	FITC-Ahx-VLRGVGDSQVPEIDRT
APN 208–223	FITC-Ahx-QSTDARKSFPCFDEPA
APN 226–244	FITC-Ahx-ATFNITLIHPNNLTALSNM
APN 367–391	FITC-Ahx-QSSSISNKERVVTVIAHELAHQWFG
APN 436–457	FITC-Ahx-YRVMAVDALASSHPLTTPAEEV
APN 469–493	FITC-Ahx-SISYSKGASVIRMLSNFLTEDLFKE
APN 553–568	FITC-Ahx-KTGNISQKHFLLDSES
APN 580–598	FITC-Ahx-WIVPISSIKNGVMQDHYWL
APN 652–670	FITC-Ahx-VINRAQVIYDSFNLATAHM
APN 841–874	FITC-Ahx-YLGYTLNPDLIRKQDATSTINSIASNVIGQPLAW
APN 901–916	FITC-Ahx-GVTRRFSSEFELQQLE
F4ab 148–160	FITC-Ahx-NASYAGVFGKGGV
F4ab 175–187	FITC-Ahx-LRAIFYGGLTTTV
F4ab 199–217	FITC-Ahx-AARTELFGSLSRNDILGQI
F4ac 149–161	FITC-Ahx-NASYAGVLGRGGV
F4ac 176–188	FITC-Ahx-LSSIFYGGLPRGS
F4ac 200–218	FITC-Ahx-TKLFGSLSRNDILGQIQRV
F4ad 149–161	FITC-Ahx-ASYAGALGRGGVT
F4ad 176–188	FITC-Ahx-HAIFYGGLPTNVK
F4ad 200–218	FITC-Ahx-ARTELFGSLSKNDILGQIQ
F4ab 148–160M	FITC-Ahx-NASYAGVFGKG*A*V
F4ac 200–218 M1	FITC-Ahx-T*A*LFGSLSRNDILGQIQRV
F4ac 200–218 M2	FITC-Ahx-TK*A*FGSLSRNDILGQIQRV
F4ac 200–218 M3	FITC-Ahx-TKLF*A*SLSRNDILGQIQRV
F4ac 200–218 M4	FITC-Ahx-TKLFGS*A*SRNDILGQIQRV
F4ac 200–218 M5	FITC-Ahx-TKLFGSL*A*RNDILGQIQRV
F4ac 200–218 M6	FITC-Ahx-TKLFGSLSR*A*DILGQIQRV
F4ac 200–218 M7	FITC-Ahx-TKLFGSLSRNDI*A*GQIQRV
F4ac 200–218 M8	FITC-Ahx-TKLFGSLSRNDIL*A*QIQRV
F4ac 200–218 M9	FITC-Ahx-TKLFGSLSRNDILGQIQ*A*V
F4ad 200–218M	FITC-Ahx-*E*RTELFGSLSKNDILGQIQ

The point-mutant amino acid in FaeG peptides are marked in italic.

### Point mutations and his pull-down assays

We introduced point mutations in the FaeG proteins using the QuikChange II XL site-directed mutagenesis kit (Agilent Technologies, USA). His-tagged APN proteins were expressed at 16 °C and loaded on a Pierce™ His protein interaction Pull-down kit (Thermo Fisher Scientific, USA) according to the manufacturer’s instructions [24]. We performed SDS-PAGE and western blotting to determine the interaction between FaeG point mutants and APN. We incubated the blots overnight in a monoclonal antibody against F4^+^ or polyclonal antibodies against APN, and stained these using enhanced chemiluminescence (ECL) (Pierce, USA) reagents.

### Ethics statement

This study was conducted in compliance with the guidelines of the Yangzhou University Institutional Animal Care and Use Committee (SYXK2016-0019). Experiments were performed in accordance with the Regulations for the Administration of Affairs Concerning Experimental Animals approved by the State Council of the People’s Republic of China.

### Tissue preparation and binding activity

We chose 25-day-old Landrace and Large White 2-way crossbred Pigs screened in our laboratory susceptible to F4 ETEC under general anesthesia using inhaled isoflurane [8, 25] and performed a midline laparotomy and exposed the proximal jejunum. We also used local anesthetic drugs to block sensation of painful. We excised the consecutive segments of the jejunum using a silk suture, with each segment of bowel measuring 5 cm. We injected a 5 mL solution with 10^−6^ mol/L FaeG peptides, 10^9^ CFU/mL rF4 bacterial strains [we introduced point mutations in SE5000 carrying PBR322-*faeG* using the QuikChange II XL site-directed mutagenesis kit (Agilent Technologies, USA)], 10^9^ CFU/mL SE5000 strain or a 10^−6^ mol/L non-binding peptide intraluminal into the targeted segment of bowel. We then took the intestine back to the abdominal cavity and sutured the cut skin. We kept the animal under general anesthesia until the intraluminal injection was completed. One hour later after the completion of the intraluminal injection, the piglet was sacrificed using CO_2_ gas and the laparotomy incision was re-opened. We removed each 5 cm segment of the jejunum and placed these in an Eppendorf tube on ice for further analysis. We cleaned the samples thrice using a cold PBS solution with 1 mg glucose and 5 mg trypsin inhibitor per milliliter.

We stored 2 cm segments of the samples in OCT (optimum cutting temperature, Sakura Finetek Japan, Tokyo) compound and rapidly froze these in liquid nitrogen, then sectioned them on a cryostat. We mounted 10 micron sections on slides with 4% paraformaldehyde, blocked with 3% BSA, and then rinsed these in PBS. After pre-incubating the tissue in an anti-F4 monoclonal antibody at 4 °C for 2 h, we rinsed the tissue using PBS, and then incubated the tissue in anti-mouse Dylight 594 secondary antibody (Univ-bio, Shanghai, China) at 37 °C for 30 min. We stained dried slides with DAPI (TianGen, Beijing, China) for 10–15 min, and then washed these prior to mounting them with cover slips, and evaluated the tissue under a Leica TCS SP8 STED confocal microscope.

### Statistical analyses

All data obtained in this study were shown as the means ± standard deviations of at least three independent samples. We statistically analyzed the relative value between the absorbance of samples and controls at a wavelength of 450 nm via GraphPad Prism^®^ 5.0 Software (GraphPad Prism Inc., CA, USA) and followed with a student’s t test using SPSS 16.0 software (SPSS Inc., USA). A *p* value of < 0.05 was regarded as significant (*), and *p* value of less than 0.01 was considered extremely significant (**).

## Results

### The APN binding domain located in the same region of the FaeG variants

The APN binding domains in the FaeG proteins were screened using overlapped peptides assays.

**Figure 1:**
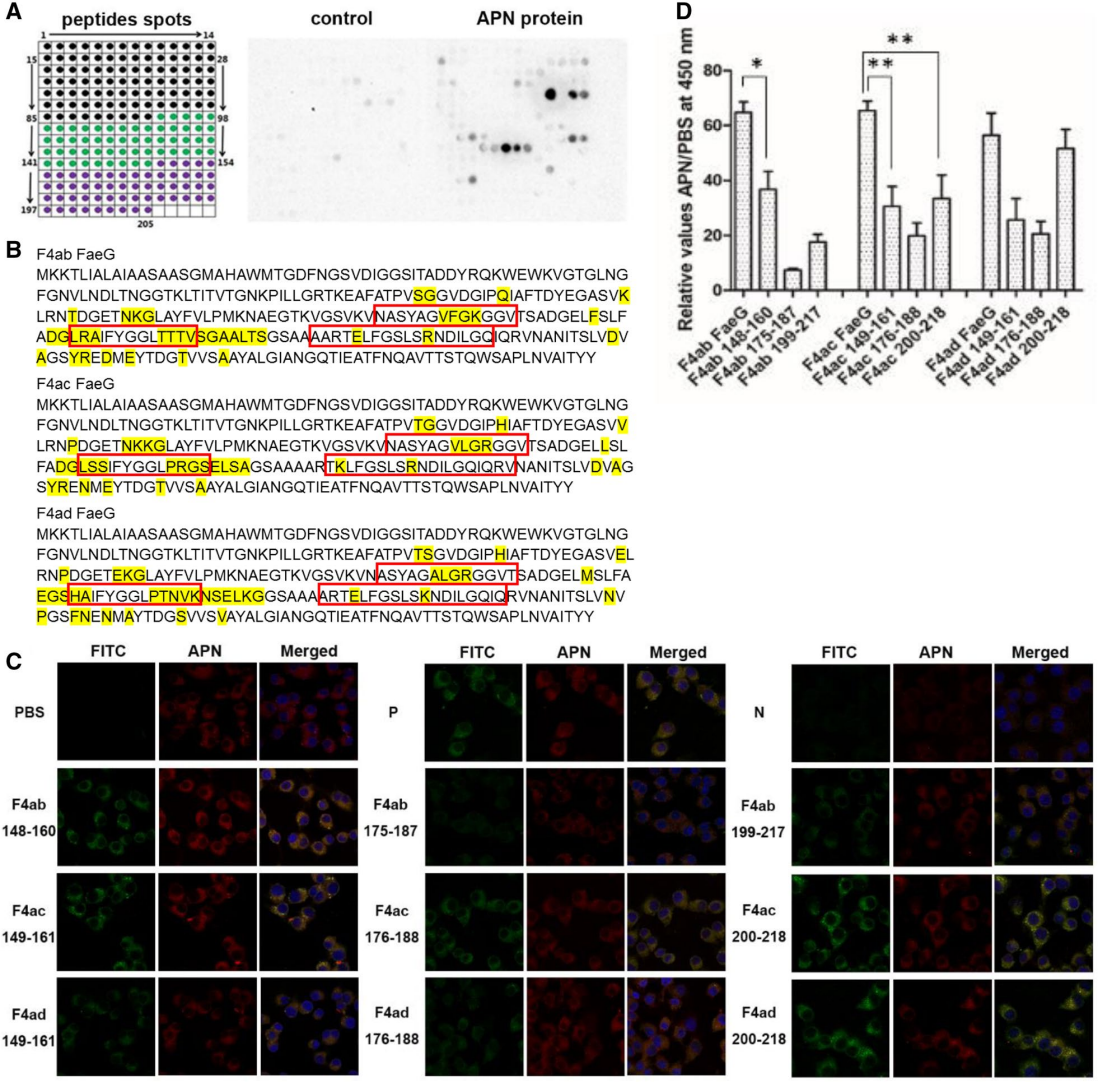
**The APN-binding sites in the FaeG protein. A** Array of overlapping FaeG peptides from the three variants (F4ab, F4ac and F4ad). Using an overlapping array of 205 peptides, we shifted three amino acids and synthesized them for the FaeG protein (Additional file 1). We detected the relative signal intensity of bound APNs to each position of the membrane by using polyclonal antibodies against the APNs. We used BSA-TBST as a negative control. Based on chemiluminescent signals we analyzed and determined the potential APN-binding sites in the FaeG. The peptide with the strongest intensity was set to 100% and all other spots were normalized to this value. We considered intensity values > 30% to be positive. **B** Amino acids analysis. The major APN-binding amino acids of the FaeG protein are positioned and marked with a red box. The differences of amino acids in the three serotypes are highlighted in yellow. **C** Confocal microscopy images. We measured how FaeG peptides interacted with APN using confocal microscopy experiments involving pEC129-APN IPEC-J2 cells and 9 overlapping peptides of FaeG (green, FITC labeled). The peptides of FaeG interacted with APN in pEC129-APN IPEC-J2 cells, the expression of APN in these cells by using polyclonal antibodies against APN and Dylight 549 goat anti-rabbit IgG secondary antibody (red), the interaction between FaeG and the pEC129-APN IPEC-J2 cell as the positive control (P) and the pcDNA™6.2-GW/miR-APN IPEC-J2 cells acted as the negative control (N). **D** ELISA assays. We coated APN proteins on ELISA plates, used 9 peptides of FaeG and the fusion FaeG proteins from three serotypes to determine the binding sites in FaeG. We repeated each experiment three times. The results shown are mean ± standard deviations (*p < 0.05, **p < 0.01).

Figure 1A shows the regions of the primary sequence of FaeG recognized by APN (indicated by the spots on the membrane). The overlapping peptides in the positive spots suggest that the APN-binding amino acids of the three variants were in the same location of the FaeG proteins (Figure 1B). We screened the AAs residues 148–160 of F4ab, 149–161, 200–218 of F4ac, and 200–218 of F4ad as the major APN-binding domains, depending on the observed strongest signals in confocal microscopy (Figure 1C). FaeG proteins showed comparable sensitivity and better specificity in APN-based ELISA tests compared to FaeG peptides. Moreover, the peptides F4ab 148–160, F4ac 200–218, and F4ad 200–218 showed better sensitivity and specificity in the APN binding compared to other FaeG peptides from the same serotype (Figure 1D).

### Critical residues of the linear epitope for APN binding

**Figure 2:**
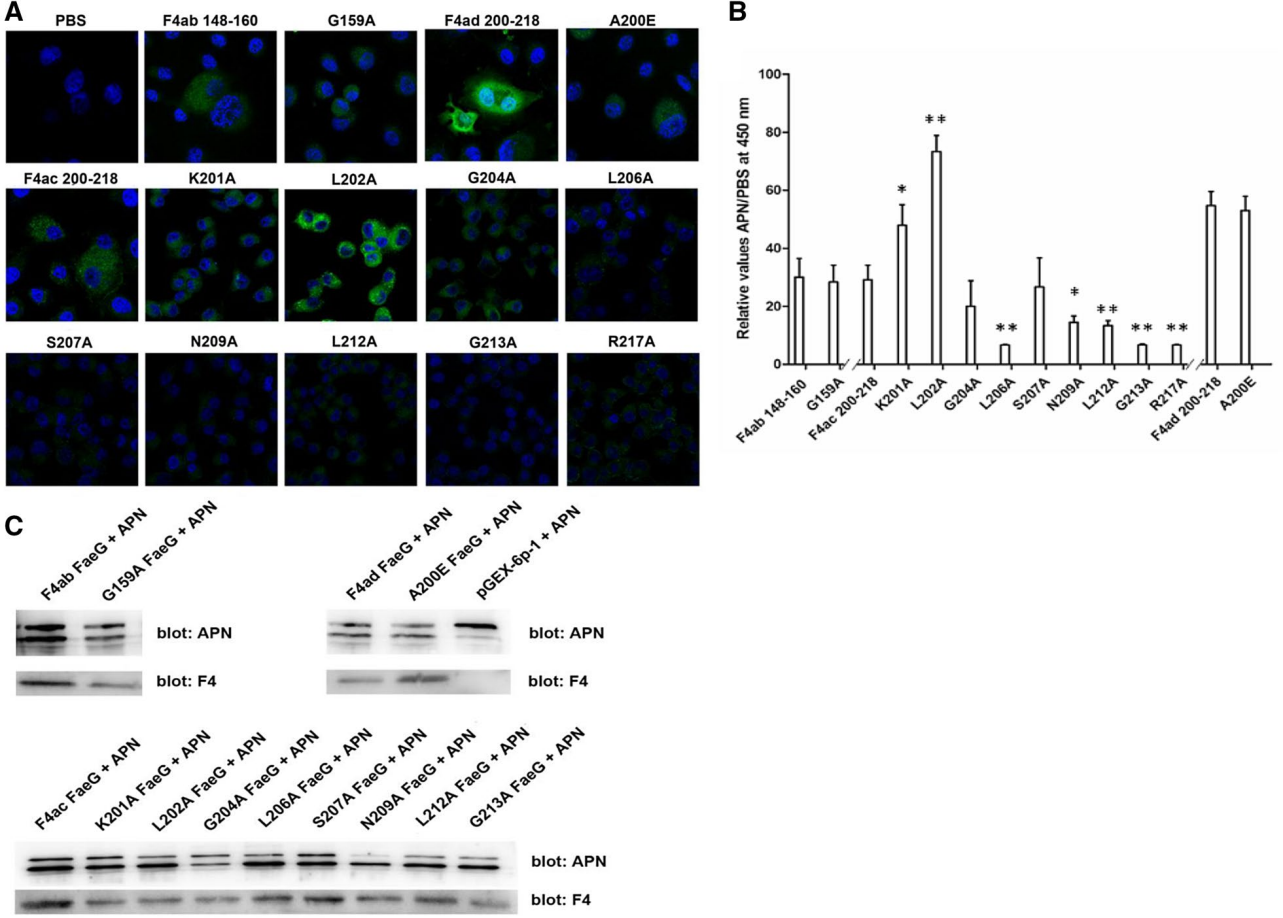
**Analysis of critical amino acids in FaeG for APN binding. A** Confocal microscopy images. We measured the critical residues of FaeG for APN binding using pEC129-APN IPEC-J2 cells and the point-mutations in the candidate FaeG peptides using confocal microscopy. **B** ELISA assays. We used the point-mutations of FaeG peptides to determine the critical residues in APN binding. We repeated the experiments thrice and data are expressed as mean ± standard deviations (*p < 0.05, **p < 0.01). **C** His pull-down assays. The APN protein is a 2-mer structure, has two bands, and is expressed at 16 °C. We studied the binding between the point-mutations of FaeG and APN proteins using the Pierce™ His protein interaction Pull-down kit. Western blotting by using anti-F4 monoclonal antiserum and anti-APN polyclonal antibodies for detection, the intensity of each band for evaluating the binding activities of APN proteins with FaeG variants.

To further analyze the core sequence motif, we used ELISA and confocal microscopy to test the single-amino acid mutants for peptides F4ab 148–160 (NASYAGVFGKGGV), F4ac 200–218 (TKLFGSLSRNDILGQIQRV), and F4ad 200–218 (ARTELFGSLSKNDILGQIQ) (Table 3). We determined that peptides with the underlined single amino acid mutation, including “NASYAGVFGKGGV” of F4ab FaeG, “TKLFGSLSRNDILGQIQRV” of F4ac FaeG, and “ARTELFGSLSKNDILGQIQ” of F4ad FaeG all had weak interactions with the APN compared to their parental peptides (Figures 2A and B). Furthermore, all of the point-mutants of the FaeG protein with a GST-tag were constructed and expressed, and G159A of F4ab, G204A, N209A, L212A, and G213A of F4ac bound weakly to His-tagged APN proteins in vitro according to the pull-down results (Figure 2C).

**Figure 3:**
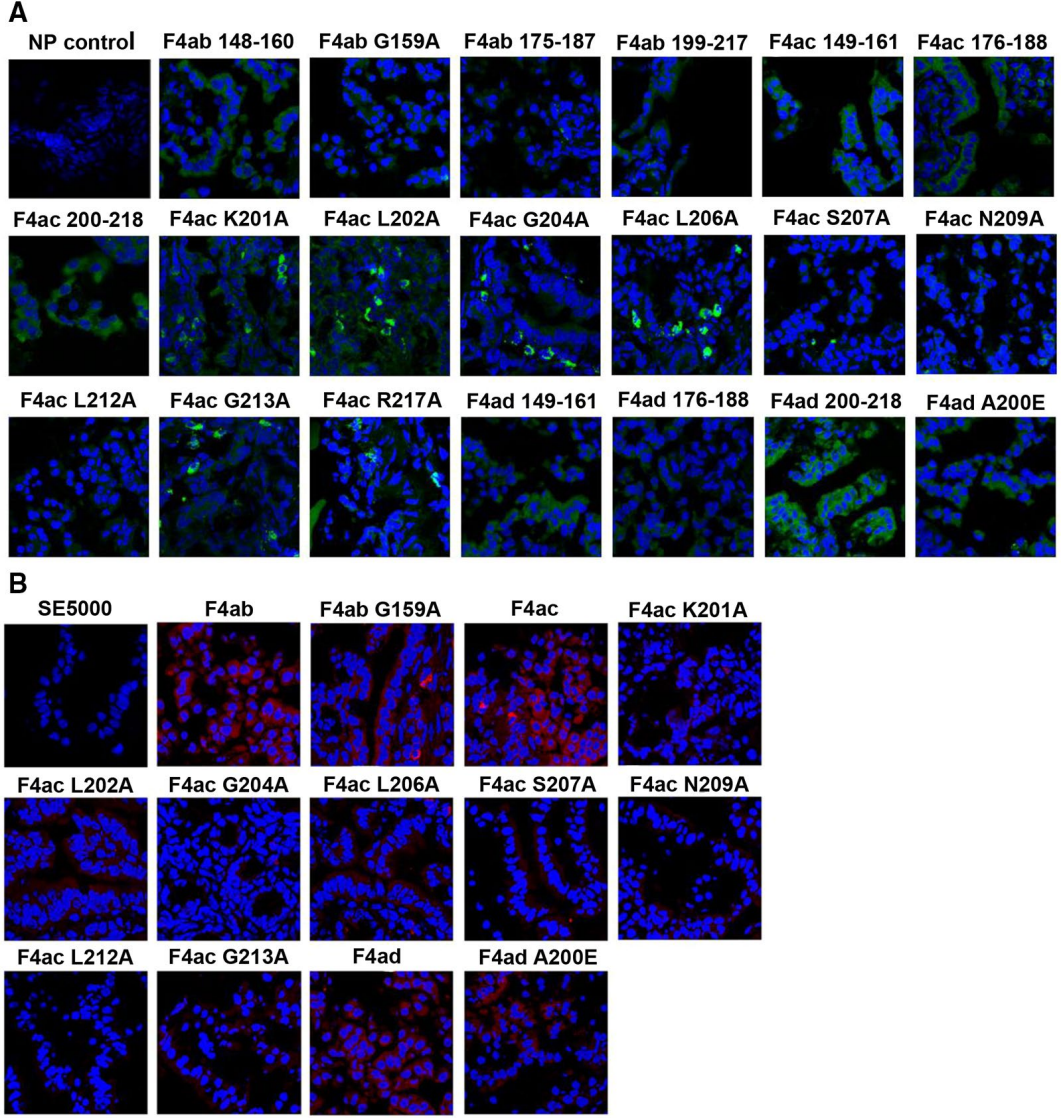
**Analysis of peptide and F4 mutant bindings to the proximal jejunal cells.** We incubated the bowel segments of the jejunum in peptides and rF4 strains (SE5000 carrying PBR322-*faeG* of F4ab, F4ac and F4ad). **A**, **B** Confocal microscopy images. We measured the binding activities of peptides with amino acid mutations (green, FITC labelled) and F4 FaeG mutants in the proximal jejunum of piglets using confocal microscopy. We tested the F4 strains in these cells by using monoclonal antibody against F4 and anti-mouse Dylight 594 secondary antibody (1:200 dilution, red). Segments with non-binding peptide (NP control) or SE5000 strain as the control.

In binding to the proximal jejunum of the piglets, confocal microscopy indicated that peptides with an amino acid mutation in G159 of F4ab, N209 and L212 of F4ac, and A200 of F4ad showed weaker staining patterns compared to their parental peptides and other peptide mutants (Figure 3A). Moreover, the FaeG mutants K201A, G204A, N209A, and L212A of F4ac exhibited similarly weak gut binding activity compared to the wild-type F4 strains, whereas G159 of F4ab, L202, L206, S207 and G213 of F4ac, and A200 of F4ad exhibited relatively weak binding reactions (Figure 3B).

### FaeG interacts with the truncated APN proteins

**Figure 4:**
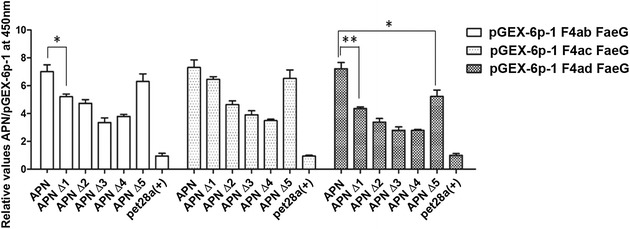
**ELISA analysis of FaeG interactions with the truncated APN proteins.** We observed the expression of five truncated APN proteins in a prokaryotic system, including APN∆1 (401–963 AA), APN∆2 (1–400 AA), APN∆3 (332–400 AA), APN∆4 (1–331 AA), and APN∆5 (332–963 AA). We analyzed the binding activity between the five purified truncated APN proteins and FaeG using indirect ELISA. pGEX-6p-1 and pet28a (+) as negative controls. We recorded the absorbance at a wavelength of 450 nm, repeated the experiments thrice. The results shown are mean ± standard deviations (*p < 0.05, **p < 0.01).

We analyzed the binding activity between each of the five purified truncated APN proteins and FaeG using indirect ELISA, and the results show that APN∆1 and APN∆5 had stronger reactivity with the FaeG protein than the other three truncated APNs; however, the whole APN protein had the strongest reaction with FaeG for all three variants (Figure 4).

### The functional motifs for FaeG binding throughout the APN protein

**Figure 5:**
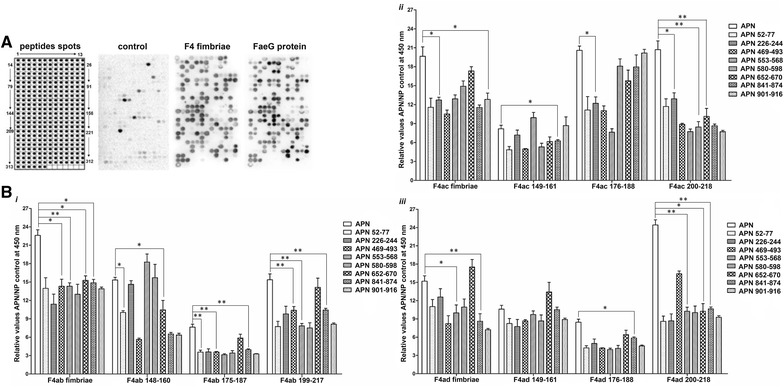
**The FaeG-binding sites in the APN protein. A** Array of overlapping APN peptides. We used a membrane spotted with peptides (Additional file 2) that spanned the entire amino acid sequence of APN for incubation in either natural F4 fimbrial proteins or fusion FaeG proteins. The image of the membrane shows the reactivity at each spot. The spot with the strongest signal are set the standard (100% intensity), all other intensity values as a relative percentage of this standard. We only considered spots with intensities > 30% as positive. **B** ELISA assays. We coated APN protein and 13 peptides of the APN on ELISA plates, and then used the coated plates to test the binding activity of the APN with the F4 fimbriae and the FaeG peptides, the APN protein as a positive control. The data presented here are the average of the three experiments and shown as mean ± standard deviations (*p < 0.05, **p < 0.01).

We determined the recognition patterns of FaeG binding to APN using the incubation between the membrane spotted with APN peptide libraries and either natural F4 fimbrial proteins or fusion FaeG proteins. Figure 5A shows an image from a representative membrane with their coordinates. We determined that sequential repetition of two or more consecutive spots on the membrane were likely to be present on the positive peptides that were involved in these interactions. The resulting 13 FaeG-binding peptides (Table 1) located throughout the APN protein and peptide APN 367–391 (AAs 367–391) were located on the zinc-binding region and encompassed a region of the truncated APN∆3 protein.

We synthesized the 13 peptides (Table 3) and coated these on ELISA plates to test the binding activity of the APN with the F4 fimbriae, as well as screened the AA residues 52–70, 226–244, 469–493, 553–568, 580–598, 652–670, 841–874 and 901–916 of the APN as the linear epitope for FaeG binding. The purified APN proteins had the most prominent binding activity compared to single or mixed APN peptides. The F4 fimbriae and FaeG peptides of all three variants vary in their ability to interact with APN peptides and protein. Apart from the AA residues 553–568 and 652–670 of APN important for FaeG binding of all three variants, the AA residues 52–70, 469–493 and 841–874 of APN are efficient for the binding with F4ab and F4ad, the AAs 901–916 are the linear epitope for F4ac, while AAs 226–244 and 580–598 are the determinant epitopes of APN to interact with F4ac and F4ad (Figure 5B, Additional file 3).

## Discussion

F4^+^ fimbriae-mediated adherence to porcine intestinal epithelial cells is an initial step in the infection process and requires that the fimbriae interact with host specific receptors [26]. Considering that FaeG directly mediates the binding of the three F4 variants to the host intestinal epithelial cells [6, 27], the only difference among the three variants involves in the *faeG* gene and results in different structural and adhesive properties to host receptors [11]. Previous studies have shown that the fimbriae of the three variants recognized and bound to different receptors, i.e., F4ab and F4ac fimbriae interacted with both sulfatide and galactosylceramide, whereas F4ad did not [28]. Moreover, previous studies have determined that residual AAs 125–163 of FaeG are essential for F4 fimbrial binding, AAs 140–145 and 151–156 are the determinant epitopes for F4ab, and 147–160 dictate the binding capacity of F4ac [29]. Additionally, AAs 150–152 and 166–170 of the F4ad FaeG subunit interact with a minimal galactose binding epitope via their D′–D″–α 1–α 2 binding domain [30], whereas APN can directly interact with FaeG in all three variants.

Porcine aminopeptidase N is a receptor for TGEV, the protein interacted with viral spike (S) glycoprotein and AAs 717–813 are essential for TGEV infectivity [31]. The presence of an N-glycosylation sequon between AAs 286–288 in porcine APN, that is absent in human APN, blocked the entry of human coronaviruses 229E. APN glycosylation determines the species specificity of group 1 coronavirus infection [32]. In addition, APN is a recently reported receptor protein for F4 fimbriae. APNs are involved in oral immune responses and clathrin-mediated endocytosis of F4 fimbriae [16]. Previous studies have found that F4 ETEC susceptibility is not associated with the genetic polymorphisms or expression differences in the APN gene, and that the α2–3,6,8 sialic acid of APN is sufficient for the binding of F4 fimbriae [16, 33]. In our previous study, we found that changes in APN expression in IPEC-J2 cells could affect F4 ETEC adherence, and the results of yeast two-hybrid and pull-down assays confirmed that APN directly binds to the major fimbrial subunit FaeG in all three variants; however, we did not think that meta-periodate (NaIO_4_) treatment had a significant impact on APN-FaeG binding in vitro [17]. We also found that the brush border membrane vesicles of F4 susceptible piglets have weaker F4 binding after 10 mM NaIO4 treatment for 30 min; this phenomenon is in accordance with earlier reports that the binding of F4ac to porcine enterocytes depends on glycosylation [16, 34]. However, details on the key glycosylation site in APN protein and whether APN glycosylation in vitro differs from that of in vivo remain unclear. Ultimately, the binding determinant in the interplay between APN and FaeG still needs to be elucidated.

In this study, we used five truncated APNs to represent different binding activities to FaeG in three variants. Both APN∆1 (401–963 AA) and APN∆5 (332–963 AA) have a stronger binding with the FaeG protein compared with other truncated APNs, so we hypothesized that the C-terminal motifs in APN proteins were essential for FaeG binding. To test this hypothesis, we used membranes spotted with APN peptides libraries incubated in both fusion FaeG proteins and natural F4 fimbrial proteins. Our data suggests that the primary sequence of APN recognized by FaeG is limited to 13 specific peptides, and that these 13 peptides are located throughout the APN protein and exhibit different binding strengths in F4ab, F4ac, and F4ad. We determined that AA residues 553–568 and 652–670 of APN are essential to all three variants.

To further enable predictions of APN binding sites in the three variants, we offset the panel of peptides representing FaeG from all three variants that we mapped onto the membrane to interact with APN proteins. We found that the APN-binding sites for the three variants were extremely similar, including AAs 148–160, 175–187, and 199–217 of F4ab, and AAs 149–161, 176–188, and 200–218 of F4ac and F4ad. However, the linear mapping might be not in accordance with the protein structure, considering fusion proteins and peptides not well glycosylated, the critical residues mentioned above might be quite different from three-dimensional (3D) images of the FaeG-APN interactions, we further adopted Modeller version 9.17 as the homology modeling tool to develop 3D structures of APN-FaeG complexes [35]. The potential interacted residues in APN-FaeG interplay were performed with the help of GRAMM-X Protein–Protein Docking Web Server v.1.2.0 [36] based on template structures obtained from the Protein Data Bank (PDB ID: APN-4FKE [37], F4ab FaeG-4WE2, F4ac FaeG-2J6R, F4ad FaeG-4WEU) (Additional file 4). Based on the stronger reactivity with APN in both ELISA and the confocal microscopy, peptides F4ab 148–160, F4ac 200–218 and F4ad 200–218 were selected for further analysis. After we confirmed the potential interacted residues of these three peptides for the APN-FaeG binding at 4Å resolution, we constructed a panel of peptides and FaeG proteins with single point mutations and used them to test for direct interplay with APN both in vitro and in vivo. We observed a major change in FaeG reactivity with mutations in the amino acids N209, L212 of F4ac FaeG, while G159 of F4ab FaeG and A200 of F4ad FaeG also effect on the APN-FaeG binding.

Our results are helpful in understanding the mechanisms of APN interactions with the three variants of FaeG. Significantly, we determined that the three variants did not have equal effectiveness in the interplay with APN. Future studies should target APNs to further investigate APN-FaeG binding characteristics and establish the differences among the three variants in ETEC infections.

## Additional files

**Additional file 1.** **Peptide spots of FaeG in the membrane.** The membranes with 205 spots cover the FaeG of the three variants (284 AAs for F4ac, and 286 AAs for both F4ab and F4ad). Spots 1 to 27 and 84 to 93 represent the common residues for all three variants, and black spots 28 to 83, green spots 94 to 149 and purple spots 150 to 205 are for the genetic variation regions of F4ab, F4ac, and F4ad, respectively.

**Additional file 2.** **Peptide spots of APN in the membrane.** The membranes with 318 spots encompass the entire APN coding region (963 AAs). Each peptide was 13 amino acids in length and offset from its neighboring peptide by 3 amino acids.

**Additional file 3.** **ELISA assays.** We coated APN protein and 13 peptides of the APN on ELISA plates, and then used the coated plates to test the binding activity of the APN with the F4 fimbriae and the FaeG peptides. We used the APN protein as a positive control. The data presented here are a supplementation for Figure 5B and shown as mean ± standard deviations.

**Additional file 4.** **Schematic representation of a portion of residues involved in docking between APN (green) and FaeG from all three variants.** The initial three-dimensional structure of APN was performed by modeller9.17 based on the template structure obtained from Protein Data Bank (PDB 4FKE). Three dimensional structures of the FaeG sequences present in the F4ab+APN (PDB 4WE2), F4ac+APN (PDB 2J6R), and F4ad+APN (PDB 4WEU) are shown as rose-red, purple, and blue in order. The potential interacted residues in APN-FaeG interplay were analyzed using PyMoL1.7.6, and the representative result at 4Å resolution is shown in this figure. The hydrogen bonds are represented by dotted lines and hydrophobic interactions are shown as sticks.
