# Supplementary material for: Binding determinants in the interplay between porcine aminopeptidase N and enterotoxigenic Escherichia coli F4 fimbriae
Source: Vet Res. 2018 Feb 26;49:23. doi: 10.1186/s13567-018-0519-9 (PMC5828407; doi:10.1186/s13567-018-0519-9)
Supplement: Supplementary file 1 — Additional file 1. Peptide spots of FaeG in the membrane. The membranes with 205 spots cover the FaeG of the three variants (284 AAs for F4ac, and 286 AAs for both F4ab and F4ad). Spots 1 to 27 and 84 to 93 represent the common residues for all three variants, and black spots 28 to 83, green spots 94 to 149 and purple spots 150 to 205 are for the genetic variation regions of F4ab, F4ac, and F4ad, respectively. [file 13567_2018_519_MOESM1_ESM.docx]

**Additional file 1** **Peptide spots of FaeG in the membrane**. The membranes with 205 spots cover the FaeG of the three variants (284 AAs for F4ac, and 286 AAs for both F4ab and F4ad). Spots 1 to 27 and 84 to 93 represent the common residues for all three variants, and black spots 28 to 83, green spots 94 to 149 and purple spots 150 to 205 are for the genetic variation regions of F4ab, F4ac, and F4ad, respectively.

| Number | Amino acids | Number | Amino acids |
| --- | --- | --- | --- |
| 1 | MKKTLIALAIAAS | 2 | TLIALAIAASAAS |
| 3 | ALAIAASAASGMA | 4 | IAASAASGMAHAW |
| 5 | SAASGMAHAWMTG | 6 | SGMAHAWMTGDFN |
| 7 | AHAWMTGDFNGSV | 8 | WMTGDFNGSVDIG |
| 9 | GDFNGSVDIGGSI | 10 | NGSVDIGGSITAD |
| 11 | VDIGGSITADDYR | 12 | GGSITADDYRQKW |
| 13 | ITADDYRQKWEWK | 14 | DDYRQKWEWKVGT |
| 15 | RQKWEWKVGTGLN | 16 | WEWKVGTGLNGFG |
| 17 | KVGTGLNGFGNVL | 18 | TGLNGFGNVLNDL |
| 19 | NGFGNVLNDLTNG | 20 | GNVLNDLTNGGTK |
| 21 | LNDLTNGGTKLTI | 22 | LTNGGTKLTITVT |
| 23 | GGTKLTITVTGNK | 24 | KLTITVTGNKPIL |
| 25 | ITVTGNKPILLGR | 26 | TGNKPILLGRTKE |
| 27 | KPILLGRTKEAFA | 28 | LLGRTKEAFATPV |
| 29 | RTKEAFATPVSGG | 30 | EAFATPVSGGVDG |
| 31 | ATPVSGGVDGIPQ | 32 | VSGGVDGIPQIAF |
| 33 | GVDGIPQIAFTDY | 34 | GIPQIAFTDYEGA |
| 35 | QIAFTDYEGASVK | 36 | FTDYEGASVKLRN |
| 37 | YEGASVKLRNTDG | 38 | ASVKLRNTDGETN |
| 39 | KLRNTDGETNKGL | 40 | NTDGETNKGLAYF |
| 41 | GETNKGLAYFVLP | 42 | NKGLAYFVLPMKN |
| 43 | LAYFVLPMKNAEG | 44 | FVLPMKNAEGTKV |
| 45 | PMKNAEGTKVGSV | 46 | NAEGTKVGSVKVN |
| 47 | GTKVGSVKVNASY | 48 | VGSVKVNASYAGV |
| 49 | VKVNASYAGVFGK | 50 | NASYAGVFGKGGV |
| 51 | YAGVFGKGGVTSA | 52 | VFGKGGVTSADGE |
| 53 | KGGVTSADGELFS | 54 | VTSADGELFSLFA |
| 55 | ADGELFSLFADGL | 56 | ELFSLFADGLRAI |
| 57 | SLFADGLRAIFYG | 58 | ADGLRAIFYGGLT |
| 59 | LRAIFYGGLTTTV | 60 | IFYGGLTTTVSGA |
| 61 | GGLTTTVSGAALT | 62 | TTTVSGAALTSG |
| 63 | VSGAALTSGSAAAS | 64 | AALTSGSAAAART |
| 65 | TSGSAAAARTELF | 66 | SAAAARTELFGSL |
| 67 | AARTELFGSLSRN | 68 | TELFGSLSRNDIL |
| 69 | FGSLSRNDILGQI | 70 | LSRNDILGQIQRV |
| 71 | NDILGQIQRVNAN | 72 | LGQIQRVNANITS |
| 73 | IQRVNANITSLVD | 74 | VNANITSLVDVAG |
| 75 | NITSLVDVAGSYR | 76 | SLVDVAGSYREDM |
| 77 | DVAGSYREDMEYT | 78 | GSYREDMEYTDGT |
| 79 | REDMEYTDGTVVS | 80 | MEYTDGTVVSAAY |
| 81 | TDGTVVSAAYALG | 82 | TVVSAAYALGIAN |
| 83 | SAAYALGIANGQT | 84 | YALGIANGQTIEA |
| 85 | GIANGQTIEATFN | 86 | NGQTIEATFNQAV |
| 87 | TIEATFNQAVTTS | 88 | ATFNQAVTTSTQW |
| 89 | NQAVTTSTQWSAP | 90 | VTTSTQWSAPLNV |
| 91 | STQWSAPLNVAIT | 92 | TQWSAPLNVAITY |
| 93 | QWSAPLNVAITYY | 94 | LGRTKEAFATPVT |
| 95 | TKEAFATPVTGGV | 96 | AFATPVTGGVDGI |
| 97 | TPVTGGVDGIPHI | 98 | TGVDGIPHIAFT |
| 99 | VDGIPHIAFTDYE | 100 | IPHIAFTDYEGAS |
| 101 | IAFTDYEGASVVL | 102 | TDYEGASVVLRNP |
| 103 | EGASVVLRNPDGE | 104 | SVVLRNPDGETNK |
| 105 | LRNPDGETNKKGL | 106 | PDGETNKKGLAYF |
| 107 | ETNKKGLAYFVLP | 108 | KKGLAYFVLPMKN |
| 109 | LAYFVLPMKNAEG | 110 | FVLPMKNAEGTKV |
| 111 | PMKNAEGTKVGSV | 112 | NAEGTKVGSVKVN |
| 113 | GTKVGSVKVNASY | 114 | VGSVKVNASYAGV |
| 115 | VKVNASYAGVLGR | 116 | NASYAGVLGRGGV |
| 117 | YAGVLGRGGVTSA | 118 | VLGRGGVTSADGE |
| 119 | RGGVTSADGELLS | 120 | VTSADGELLSLFA |
| 121 | ADGELLSLFADGL | 122 | ELLSLFADGLSSI |
| 123 | SLFADGLSSIFYG | 124 | ADGLSSIFYGGLP |
| 125 | LSSIFYGGLPRGS | 126 | IFYGGLPRGSELS |
| 127 | GGLPRGSELSAGS | 128 | PRGSELSAGSAAA |
| 129 | SELSAGSAAAART | 130 | SAGSAAAARTKLF |
| 131 | SAAAARTKLFGSL | 132 | AARTKLFGSLSRN |
| 133 | TKLFGSLSRNDIL | 134 | FGSLSRNDILGQI |
| 135 | LSRNDILGQIQRV | 136 | NDILGQIQRVNAN |
| 137 | LGQIQRVNANITS | 138 | IQRVNANITSLVD |
| 139 | VNANITSLVDVAG | 140 | NITSLVDVAGSYR |
| 141 | SLVDVAGSYRENM | 142 | DVAGSYRENMEYT |
| 143 | GSYRENMEYTDGT | 144 | RENMEYTDGTVVS |
| 145 | MEYTDGTVVSAAY | 146 | TDGTVVSAAYALG |
| 147 | TVVSAAYALGIAN | 148 | SAAYALGIANGQT |
| 149 | AAYALGIANGQTI | 150 | LGRTKEAFATPVT |
| 151 | TKEAFATPVTSGV | 152 | AFATPVTSGVDGI |
| 153 | TPVTSGVDGIPHI | 154 | TSGVDGIPHIAFT |
| 155 | VDGIPHIAFTDYE | 156 | IPHIAFTDYEGAS |
| 157 | IAFTDYEGASVEL | 158 | TDYEGASVELRNP |
| 159 | EGASVELRNPDGE | 160 | SVELRNPDGETEK |
| 161 | LRNPDGETEKGLA | 162 | PDGETEKGLAYFV |
| 163 | ETEKGLAYFVLPM | 164 | KGLAYFVLPMKNA |
| 165 | AYFVLPMKNAEGT | 166 | VLPMKNAEGTKVG |
| 167 | MKNAEGTKVGSVK | 168 | AEGTKVGSVKVNA |
| 169 | TKVGSVKVNASYA | 170 | GSVKVNASYAGAL |
| 171 | KVNASYAGALGRG | 172 | ASYAGALGRGGVT |
| 173 | AGALGRGGVTSAD | 174 | LGRGGVTSADGEL |
| 175 | GGVTSADGELMSL | 176 | TSADGELMSLFAE |
| 177 | DGELMSLFAEGSH | 178 | LMSLFAEGSHAIF |
| 179 | LFAEGSHAIFYGG | 180 | EGSHAIFYGGLPT |
| 181 | HAIFYGGLPTNVK | 182 | FYGGLPTNVKNSE |
| 183 | GLPTNVKNSELKG | 184 | TNVKNSELKGGSA |
| 185 | KNSELKGGSAAAA | 186 | ELKGGSAAAARTE |
| 187 | GGSAAAARTELFG | 188 | AAAARTELFGSLS |
| 189 | ARTELFGSLSKND | 190 | ELFGSLSKNDILG |
| 191 | GSLSKNDILGQIQ | 192 | SKNDILGQIQRVN |
| 193 | DILGQIQRVNANI | 194 | GQIQRVNANITSL |
| 195 | QRVNANITSLVNV | 196 | NANITSLVNVPGS |
| 197 | ITSLVNVPGSFNE | 198 | LVNVPGSFNENMA |
| 199 | VPGSFNENMAYTD | 200 | SFNENMAYTDGSV |
| 201 | ENMAYTDGSVVSV | 202 | AYTDGSVVSVAYA |
| 203 | DGSVVSVAYALGI | 204 | VVSVAYALGIANG |
| 205 | VAYALGIANGQTI |  |  |
