# Supplementary material for: Binding determinants in the interplay between porcine aminopeptidase N and enterotoxigenic Escherichia coli F4 fimbriae
Source: Vet Res. 2018 Feb 26;49:23. doi: 10.1186/s13567-018-0519-9 (PMC5828407; doi:10.1186/s13567-018-0519-9)
Supplement: Supplementary file 2 — Additional file 2. Peptide spots of APN in the membrane. The membranes with 318 spots encompass the entire APN coding region (963 AAs). Each peptide was 13 amino acids in length and offset from its neighboring peptide by 3 amino acids. [file 13567_2018_519_MOESM2_ESM.docx]

**Additional file 2** **Peptide spots of APN in the membrane.** The membranes with 318 spots encompass the entire APN coding region (963 AAs). Each peptide was 13 amino acids in length and offset from its neighboring peptide by 3 amino acids.

| Number | Amino acids | Number | Amino acids |
| --- | --- | --- | --- |
| 1 | MAKGFYISKALGI | 2 | GFYISKALGILGI |
| 3 | ISKALGILGILLG | 4 | ALGILGILLGVAA |
| 5 | ILGILLGVAAVAT | 6 | ILLGVAAVATIIA |
| 7 | GVAAVATIIALSV | 8 | AVATIIALSVVYA |
| 9 | TIIALSVVYAQEK | 10 | ALSVVYAQEKNKN |
| 11 | VVYAQEKNKNAEH | 12 | AQEKNKNAEHVPQ |
| 13 | KNKNAEHVPQAPT | 14 | NAEHVPQAPTSPT |
| 15 | HVPQAPTSPTITT | 16 | QAPTSPTITTTAA |
| 17 | TSPTITTTAAITL | 18 | TITTTAAITLDQS |
| 19 | TTAAITLDQSKPW | 20 | AITLDQSKPWNRY |
| 21 | LDQSKPWNRYRLP | 22 | SKPWNRYRLPTTL |
| 23 | WNRYRLPTTLLPD | 24 | YRLPTTLLPDSYF |
| 25 | PTTLLPDSYFVTL | 26 | LLPDSYFVTLRPY |
| 27 | DSYFVTLRPYLTP | 28 | FVTLRPYLTPNAD |
| 29 | LRPYLTPNADGLY | 30 | YLTPNADGLYIFK |
| 31 | PNADGLYIFKGKS | 32 | DGLYIFKGKSIVR |
| 33 | YIFKGKSIVRLLC | 34 | KGKSIVRLLCQEP |
| 35 | SIVRLLCQEPTDV | 36 | RLLCQEPTDVIII |
| 37 | CQEPTDVIIIHSK | 38 | PTDVIIIHSKKLN |
| 39 | VIIIHSKKLNYTT | 40 | IHSKKLNYTTQGH |
| 41 | KKLNYTTQGHMVV | 42 | NYTTQGHMVVLRG |
| 43 | TQGHMVVLRGVGD | 44 | HMVVLRGVGDSQV |
| 45 | VLRGVGDSQVPEI | 46 | GVGDSQVPEIDRT |
| 47 | DSQVPEIDRTELV | 48 | VPEIDRTELVELT |
| 49 | IDRTELVELTEYL | 50 | TELVELTEYLVVH |
| 51 | VELTEYLVVHLKG | 52 | TEYLVVHLKGSLQ |
| 53 | LVVHLKGSLQPGH | 54 | HLKGSLQPGHMYE |
| 55 | GSLQPGHMYEMES | 56 | QPGHMYEMESEFQ |
| 57 | HMYEMESEFQGEL | 58 | EMESEFQGELADD |
| 59 | SEFQGELADDLAG | 60 | QGELADDLAGFYR |
| 61 | LADDLAGFYRSEY | 62 | DLAGFYRSEYMEG |
| 63 | GFYRSEYMEGNVK | 64 | RSEYMEGNVKKVL |
| 65 | YMEGNVKKVLATT | 66 | GNVKKVLATTQMQ |
| 67 | KKVLATTQMQSTD | 68 | LATTQMQSTDARK |
| 69 | TQMQSTDARKSFP | 70 | QSTDARKSFPCFD |
| 71 | DARKSFPCFDEPA | 72 | KSFPCFDEPAMKA |
| 73 | PCFDEPAMKATFN | 74 | DEPAMKATFNITL |
| 75 | AMKATFNITLIHP | 76 | ATFNITLIHPNNL |
| 77 | NITLIHPNNLTAL | 78 | LIHPNNLTALSNM |
| 79 | PNNLTALSNMPPK | 80 | LTALSNMPPKGSS |
| 81 | LSNMPPKGSSTPL | 82 | MPPKGSSTPLAED |
| 83 | KGSSTPLAEDPNW | 84 | STPLAEDPNWSVT |
| 85 | LAEDPNWSVTEFE | 86 | DPNWSVTEFETTP |
| 87 | WSVTEFETTPVMS | 88 | TEFETTPVMSTYL |
| 89 | ETTPVMSTYLLAY | 90 | PVMSTYLLAYIVS |
| 91 | STYLLAYIVSEFQ | 92 | LLAYIVSEFQSVN |
| 93 | YIVSEFQSVNETA | 94 | SEFQSVNETAQNG |
| 95 | QSVNETAQNGVLI | 96 | NETAQNGVLIRIW |
| 97 | AQNGVLIRIWARP | 98 | GVLIRIWARPNAI |
| 99 | IRIWARPNAIAEG | 100 | WARPNAIAEGHGM |
| 101 | PNAIAEGHGMYAL | 102 | IAEGHGMYALNVT |
| 103 | GHGMYALNVTGPI | 104 | MYALNVTGPILNF |
| 105 | LNVTGPILNFFAN | 106 | TGPILNFFANHYN |
| 107 | ILNFFANHYNTSY | 108 | FFANHYNTSYPLP |
| 109 | NHYNTSYPLPKSD | 110 | NTSYPLPKSDQIA |
| 111 | YPLPKSDQIALPD | 112 | PKSDQIALPDFNA |
| 113 | DQIALPDFNAGAM | 114 | ALPDFNAGAMENW |
| 115 | DFNAGAMENWGLV | 116 | AGAMENWGLVTYR |
| 117 | MENWGLVTYRENA | 118 | WGLVTYRENALLF |
| 119 | VTYRENALLFDPQ | 120 | RENALLFDPQSSS |
| 121 | ALLFDPQSSSISN | 122 | FDPQSSSISNKER |
| 123 | QSSSISNKERVVT | 124 | SISNKERVVTVIA |
| 125 | NKERVVTVIAHEL | 126 | RVVTVIAHELAHQ |
| 127 | TVIAHELAHQWFG | 128 | AHELAHQWFGNLV |
| 129 | LAHQWFGNLVTLA | 130 | QWFGNLVTLAWWN |
| 131 | GNLVTLAWWNDLW | 132 | VTLAWWNDLWLNE |
| 133 | AWWNDLWLNEGFA | 134 | NDLWLNEGFASYV |
| 135 | WLNEGFASYVEYL | 136 | EGFASYVEYLGAD |
| 137 | ASYVEYLGADHAE | 138 | VEYLGADHAEPTW |
| 139 | LGADHAEPTWNLK | 140 | DHAEPTWNLKDLI |
| 141 | EPTWNLKDLIVPG | 142 | WNLKDLIVPGDVY |
| 143 | KDLIVPGDVYRVM | 144 | IVPGDVYRVMAVD |
| 145 | GDVYRVMAVDALA | 146 | YRVMAVDALASSH |
| 147 | MAVDALASSHPLT | 148 | DALASSHPLTTPA |
| 149 | ASSHPLTTPAEEV | 150 | HPLTTPAEEVNTP |
| 151 | TTPAEEVNTPAQI | 152 | AEEVNTPAQISEM |
| 153 | VNTPAQISEMFDS | 154 | PAQISEMFDSISY |
| 155 | ISEMFDSISYSKG | 156 | MFDSISYSKGASV |
| 157 | SISYSKGASVIRM | 158 | YSKGASVIRMLSN |
| 159 | GASVIRMLSNFLT | 160 | VIRMLSNFLTEDL |
| 161 | MLSNFLTEDLFKE | 162 | NFLTEDLFKEGLA |
| 163 | TEDLFKEGLASYL | 164 | LFKEGLASYLHAF |
| 165 | EGLASYLHAFAYQ | 166 | ASYLHAFAYQNTT |
| 167 | LHAFAYQNTTYLD | 168 | FAYQNTTYLDLWE |
| 169 | QNTTYLDLWEHLQ | 170 | TYLDLWEHLQKAV |
| 171 | DLWEHLQKAVDAQ | 172 | EHLQKAVDAQTSI |
| 173 | QKAVDAQTSIRLP | 174 | VDAQTSIRLPDTV |
| 175 | QTSIRLPDTVRAI | 176 | IRLPDTVRAIMDR |
| 177 | PDTVRAIMDRWTL | 178 | VRAIMDRWTLQMG |
| 179 | IMDRWTLQMGFPV | 180 | RWTLQMGFPVITV |
| 181 | LQMGFPVITVDTK | 182 | GFPVITVDTKTGN |
| 183 | VITVDTKTGNISQ | 184 | VDTKTGNISQKHF |
| 185 | KTGNISQKHFLLD | 186 | NISQKHFLLDSES |
| 187 | QKHFLLDSESNVT | 188 | FLLDSESNVTRSS |
| 189 | DSESNVTRSSAFD | 190 | SNVTRSSAFDYLW |
| 191 | TRSSAFDYLWIVP | 192 | SAFDYLWIVPISS |
| 193 | DYLWIVPISSIKN | 194 | WIVPISSIKNGVM |
| 195 | PISSIKNGVMQDH | 196 | SIKNGVMQDHYWL |
| 197 | NGVMQDHYWLRDV | 198 | MQDHYWLRDVSQA |
| 199 | HYWLRDVSQAQND | 200 | LRDVSQAQNDLFK |
| 201 | VSQAQNDLFKTAS | 202 | AQNDLFKTASDDW |
| 203 | DLFKTASDDWVLL | 204 | KTASDDWVLLNVN |
| 205 | SDDWVLLNVNVTG | 206 | WVLLNVNVTGYFQ |
| 207 | LNVNVTGYFQVNY | 208 | NVTGYFQVNYDED |
| 209 | GYFQVNYDEDNWR | 210 | QVNYDEDNWRMIQ |
| 211 | YDEDNWRMIQHQL | 212 | DNWRMIQHQLQTN |
| 213 | RMIQHQLQTNLSV | 214 | QHQLQTNLSVIPV |
| 215 | LQTNLSVIPVINR | 216 | NLSVIPVINRAQV |
| 217 | VIPVINRAQVIYD | 218 | VINRAQVIYDSFN |
| 219 | RAQVIYDSFNLAT | 220 | VIYDSFNLATAHM |
| 221 | DSFNLATAHMVPV | 222 | NLATAHMVPVTLA |
| 223 | TAHMVPVTLALDN | 224 | MVPVTLALDNTLF |
| 225 | VTLALDNTLFLNG | 226 | ALDNTLFLNGEKE |
| 227 | NTLFLNGEKEYMP | 228 | FLNGEKEYMPWQA |
| 229 | GEKEYMPWQAALS | 230 | EYMPWQAALSSLS |
| 231 | PWQAALSSLSYFS | 232 | AALSSLSYFSLMF |
| 233 | SSLSYFSLMFDRS | 234 | SYFSLMFDRSEVY |
| 235 | SLMFDRSEVYGPM | 236 | FDRSEVYGPMKKY |
| 237 | SEVYGPMKKYLRK | 238 | YGPMKKYLRKQVE |
| 239 | MKKYLRKQVEPLF | 240 | YLRKQVEPLFQHF |
| 241 | KQVEPLFQHFETL | 242 | EPLFQHFETLTKN |
| 243 | FQHFETLTKNWTE | 244 | FETLTKNWTERPE |
| 245 | LTKNWTERPENLM | 246 | NWTERPENLMDQY |
| 247 | ERPENLMDQYSEI | 248 | ENLMDQYSEINAI |
| 249 | MDQYSEINAISTA | 250 | YSEINAISTACSN |
| 251 | INAISTACSNGLP | 252 | ISTACSNGLPQCE |
| 253 | ACSNGLPQCENA | 254 | NGLPQCENLAKTL |
| 255 | PQCENLAKTLFDQ | 256 | ENLAKTLFDQWMS |
| 257 | AKTLFDQWMSDPE | 258 | LFDQWMSDPENNP |
| 259 | QWMSDPENNPIHP | 260 | SDPENNPIHPNLR |
| 261 | ENNPIHPNLRSTI | 262 | PIHPNLRSTIYCN |
| 263 | PNLRSTIYCNAIA | 264 | RSTIYCNAIAQGG |
| 265 | IYCNAIAQGGQDQ | 266 | NAIAQGGQDQWDF |
| 267 | AQGGQDQWDFAWG | 268 | GQDQWDFAWGQLQ |
| 269 | QWDFAWGQLQQAQ | 270 | FAWGQLQQAQLVN |
| 271 | GQLQQAQLVNEAD | 272 | QQAQLVNEADKLR |
| 273 | QLVNEADKLRSAL | 274 | NEADKLRSALACS |
| 275 | DKLRSALACSNEV | 276 | RSALACSNEVWLL |
| 277 | LACSNEVWLLNRY | 278 | SNEVWLLNRYLGY |
| 279 | VWLLNRYLGYTLN | 280 | LNRYLGYTLNPDL |
| 281 | YLGYTLNPDLIRK | 282 | YTLNPDLIRKQDA |
| 283 | NPDLIRKQDATST | 284 | LIRKQDATSTINS |
| 285 | KQDATSTINSIAS | 286 | ATSTINSIASNVI |
| 287 | TINSIASNVIGQP | 288 | SIASNVIGQPLAW |
| 289 | SNVIGQPLAWDFV | 290 | IGQPLAWDFVQSN |
| 291 | PLAWDFVQSNWKK | 292 | WDFVQSNWKKLFQ |
| 293 | VQSNWKKLFQDYG | 294 | NWKKLFQDYGGGS |
| 295 | KLFQDYGGGSFSF | 296 | QDYGGGSFSFSNL |
| 297 | GGGSFSFSNLIQG | 298 | SFSFSNLIQGVTR |
| 299 | FSNLIQGVTRRFS | 300 | LIQGVTRRFSSEF |
| 301 | GVTRRFSSEFELQ | 302 | RRFSSEFELQQLE |
| 303 | SSEFELQQLEQFK | 304 | FELQQLEQFKKNN |
| 305 | QQLEQFKKNNMDV | 306 | EQFKKNNMDVGFG |
| 307 | KKNNMDVGFGSGT | 308 | NMDVGFGSGTRAL |
| 309 | VGFGSGTRALEQA | 310 | GSGTRALEQALEK |
| 311 | TRALEQALEKTKA | 312 | LEQALEKTKANIK |
| 313 | ALEKTKANIKWVK | 314 | KTKANIKWVKENK |
| 315 | ANIKWVKENKEVV | 316 | KWVKENKEVVLNW |
| 317 | KENKEVVLNWFIE | 318 | NKEVVLNWFIEHS |
