# Supplementary figures and images for: Binding determinants in the interplay between porcine aminopeptidase N and enterotoxigenic Escherichia coli F4 fimbriae
Source: Vet Res. 2018 Feb 26;49:23. doi: 10.1186/s13567-018-0519-9 (PMC5828407; doi:10.1186/s13567-018-0519-9)

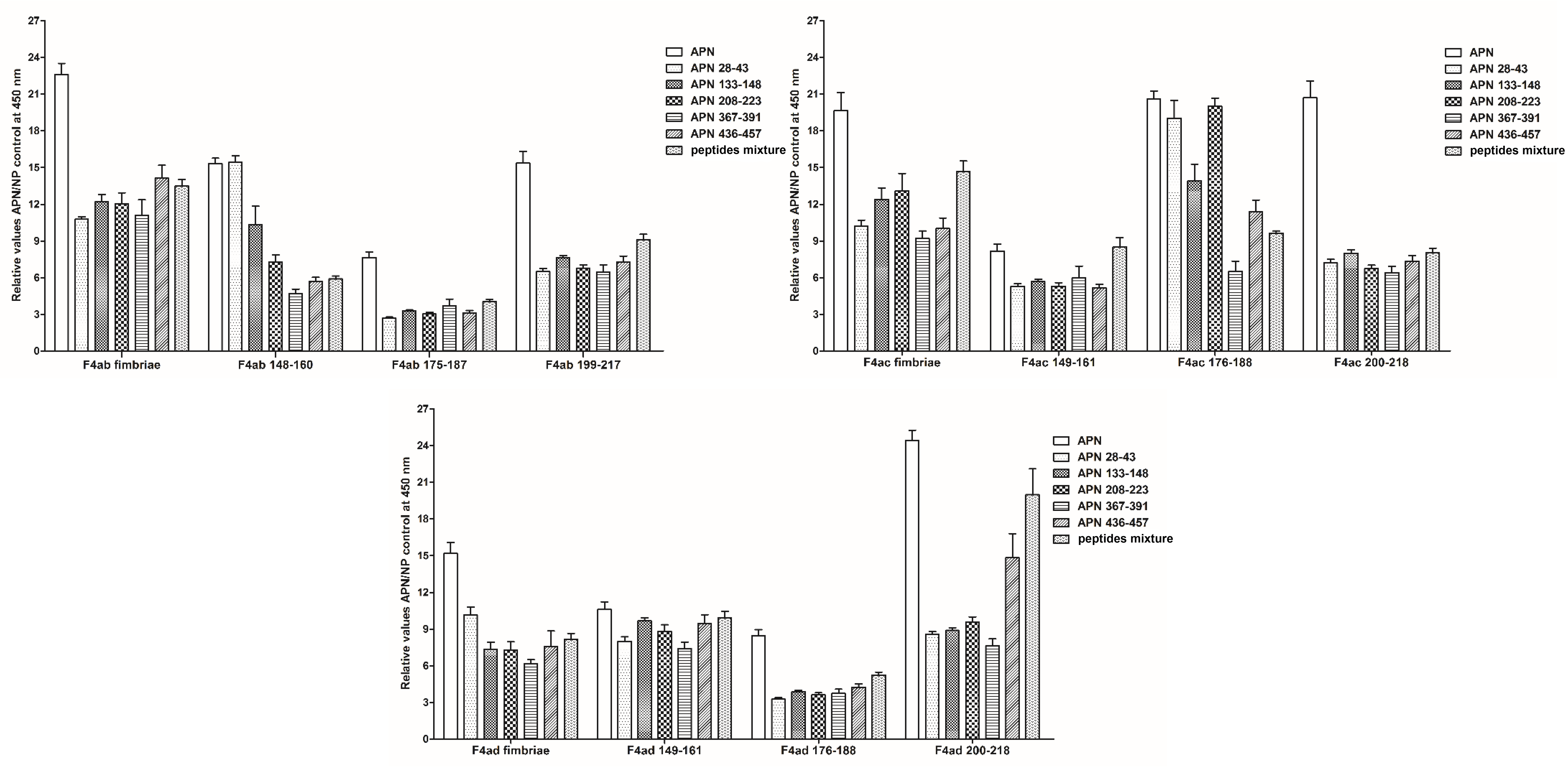

Supplement: Supplementary file 3 — Additional file 3. ELISA assays. We coated APN protein and 13 peptides of the APN on ELISA plates, and then used the coated plates to test the binding activity of the APN with the F4 fimbriae and the FaeG peptides. We used the APN protein as a positive control. The data presented here are a supplementation for Figure 5B and shown as mean ± standard deviations. [file 13567_2018_519_MOESM3_ESM.tif]

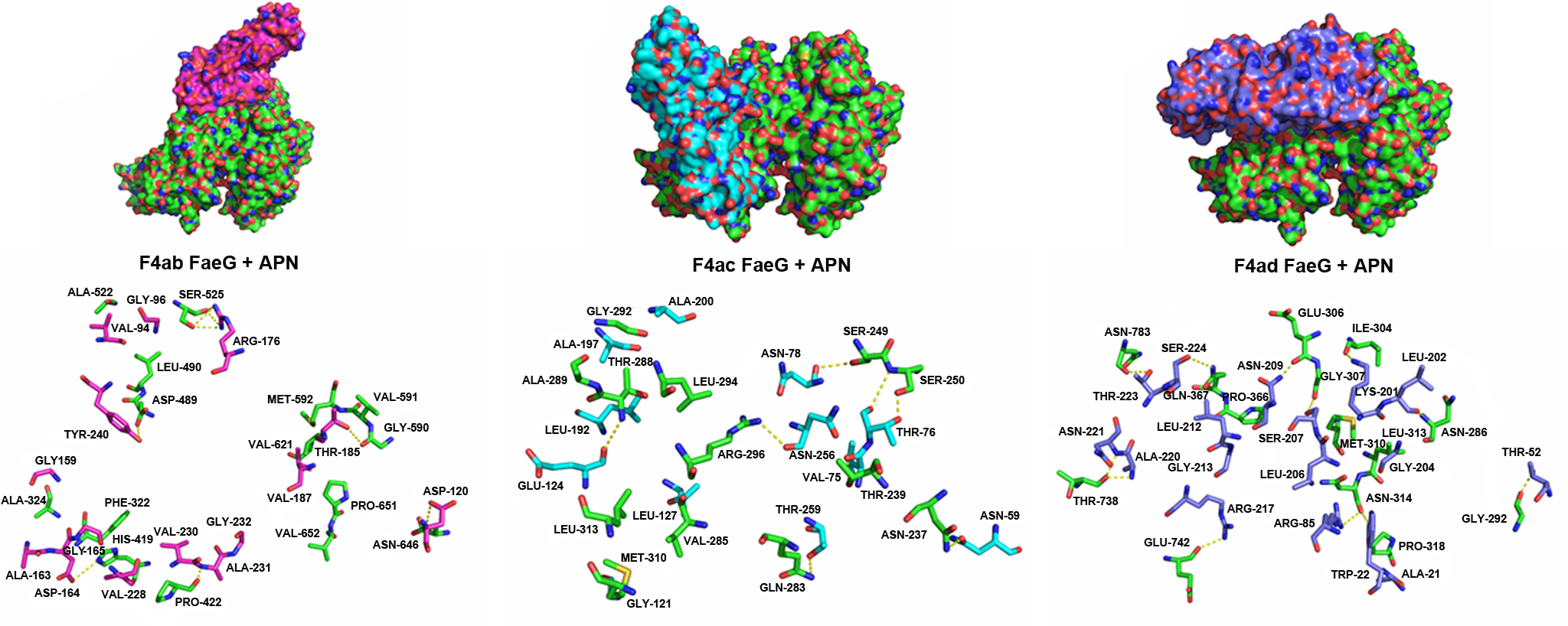

Supplement: Supplementary file 4 — Additional file 4. Schematic representation of a portion of residues involved in docking between APN (green) and FaeG from all three variants. The initial three-dimensional structure of APN was performed by modeller9.17 based on the template structure obtained from Protein Data Bank (PDB 4FKE). Three dimensional structures of the FaeG sequences present in the F4ab+APN (PDB 4WE2), F4ac+APN (PDB 2J6R), and F4ad+APN (PDB 4WEU) are shown as rose-red, purple, and blue in order. The potential interacted residues in APN-FaeG interplay were analyzed using PyMoL1.7.6, and the representative result at 4Å resolution is shown in this figure. The hydrogen bonds are represented by dotted lines and hydrophobic interactions are shown as sticks. [file 13567_2018_519_MOESM4_ESM.tif]
